# Sieve-Like CNT Film Coupled with TiO_2_ Nanowire for High-Performance Continuous-Flow Photodegradation of Rhodamine B under Visible Light Irradiation

**DOI:** 10.3390/nano11051335

**Published:** 2021-05-19

**Authors:** Zhengpeng Yang, Xiaoting Lv, Xuqing Liu, Shengmin Jia, Yongyi Zhang, Yingying Yu, Chunjing Zhang, Dandan Liu

**Affiliations:** 1 Henan Key Laboratory of Materials on Deep-Earth Engineering, School of Materials Science and Engineering, Henan Polytechnic University, Jiaozuo 454003, China; zhengpengyang2013@163.com (Z.Y.); 18339113590@163.com (X.L.); smjia2019@sinano.ac.cn (S.J.); chunjingzhang2013@163.com (C.Z.); 2 Key Laboratory of Multifunctional Nanomaterials and Smart Systems, Advanced Materials Division, Suzhou Institute of Nano-Tech and Nano-Bionics, Chinese Academy of Sciences, Suzhou 215123, China; 3 Department of Materials, The University of Manchester, Oxford Road, Manchester M13 9PL, UK; xuqing.liu@manchester.ac.uk; 4 Jiangxi Key Lab of Carbonene Materials, Division of Nanomaterials, Suzhou Institute of Nano-Tech and Nano-Bionics, Chinese Academy of Sciences, Nanchang 330200, China

**Keywords:** sieve-like CNT film, TiO_2_ nanowire, continuous-flow device, photodegradation, pollutant

## Abstract

Continuous-flow photoreactors hold great promise for the highly efficient photodegradation of pollutants due to their continuity and sustainability. However, how to enable a continuous-flow photoreactor with the combined features of high photodegradation efficiency and durability as well as broad-wavelength light absorption and large-scale processing remains a significant challenge. Herein, we demonstrate a facile and effective strategy to construct a sieve-like carbon nanotube (CNT)/TiO_2_ nanowire film (SCTF) with superior flexibility (180° bending), high tensile strength (75–82 MPa), good surface wettability, essential light penetration and convenient visible light absorption. Significantly, the unique architecture, featuring abundant, well-ordered and uniform mesopores with ca. 70 µm in diameter, as well as a homogenous distribution of TiO_2_ nanowires with an average diameter of ca. 500 nm, could act as a “waterway” for efficient solution infiltration through the SCTF, thereby, enabling the photocatalytic degradation of polluted water in a continuous-flow mode. The optimized SCTF-2.5 displayed favorable photocatalytic behavior with 96% degradation of rhodamine B (RhB) within 80 min and a rate constant of 0.0394 min^−1^. The continuous-flow photodegradation device made using SCTF-2.5 featured exceptional photocatalytic behavior for the continuous degradation of RhB under simulated solar irradiation with a high degradation ratio (99.6%) and long-term stability (99.2% retention after working continuously for 72 h). This work sheds light on new strategies for designing and fabricating high-performance continuous-flow photoreactors toward future uses.

## 1. Introduction

The emergence of continuous-flow devices opens an avenue to boost the prosperous development of successive batch manufacturing and has triggered a tremendous upsurge for the potential application in a variety of fields, such as chemical synthesis [1], mixture separation [2], pollutant treatment [3] and fuel cells [4], owing to the advantageous features of high efficiency, easy scalability, simple operation and low cost [5,6]. Of particular interest, the developing continuous-flow photoreactors, which use light for energy input, are increasingly appreciated as a reliable means to leap over the restrictions of traditional intermittent photocatalytic platforms and are regarded as a more promising strategy to carry out the photocatalytic removal of pollutants due to their continuity and sustainability [7,8].

To date, these materials with prominent photocatalytic properties, such as Sq-azo@PMO [9], ZnO-SnO2@C [10], anatase TiO2 [11], Bi2WO6/ZnFe2O4 [12], B/g-C3N4 [13], Ag3PO4/g-C3N4 [14] and UiO-66(Ti)-Fe3O4-WO3 [15], have been intensively explored for constructing continuous-flow photoreactors. Despite the exciting progress made in continuous-flow photoreactors for the photodegradation of pollutants, several issues remain to be addressed to improve the photodegradation efficiency, broad-wavelength light absorption, durability and large-scale processing to meet the rapidly growing demand in practical applications. Consequently, exploring and achieving photocatalytic materials and their rational structures that are well-matched to the continuous-flow mode for acquiring better photodegradation performances is highly desirable yet greatly challenging thus far.

CNT film, a macroscopic assembly of nanoscale individual CNTs, can be fabricated by various approaches, such as floating catalyst chemical vapor deposition (FCCVD) [16,17,18], dry spinning [19], solution-based coating [20], printing [21] and vacuum filtration [22], with different types of architectures, including non-woven cloth, vertical alignment, aligned networks, transparent film, random planar film and compressible foams [23]. 

Owing to its prominent flexibility, high mechanical strength, electrical conductivity, ease of processing, good optical transparency, prominent durability and stability, superior abrasion resistance, good adhesion properties with many substrates and low density [24,25], CNT films have been extensively applied to various fields, including transparent conductor [26], sensor [27], energy storage and conversion [28,29], filtration membrane [30], transistor [31], integrated circuits [32], etc. Significantly, the multifunctionalization of CNT film can be readily realized through combinations with various active and passive components (e.g., metal nanoparticles and nanowires, metal oxides, conductive polymers and graphene) [33]. 

Additionally, the flexible and robust CNT film fabricated by the FCCVD method could be processed into porous architecture with ample well-ordered and uniform mesopores via a facile and effective laser drilling, which was used as a smart and flexible supercapacitor [34]. Thus, after its functionalization with photoactive materials, the mesoporous CNT film can be converted into a hybrid film that combines the best features of both components, i.e., superior flexibility, mechanical strength, photocatalytic capacity, stability and processibility, which might serve as suitable scaffolds for the continuous-flow photodegradation of polluted water.

Proving this hypothesis, we designed and fabricated a flexible and robust sieve-like carbon nanotube (CNT)/TiO2 nanowire film (SCTF) for the continuous-flow removal of RhB used as a model pollutant with a laser-drilling CNT film coupled with TiO2 nanowire. The resulting SCTF contained abundant, well-ordered and uniform mesopores, thereby, enabling channels for efficient solution infiltration through the film. The good transmittance of the as-prepared hybrid film not only facilitated light penetration into the film interior but also favored effective visible light absorption. Benefiting from these unique features, the assembled continuous-flow photodegradation device based on SCTF displayed superior photocatalytic behavior for the continuous degradation of RhB under simulated solar irradiation in terms of the degradation ratio and long-term stability.

## 2. Experimental

### 2.1. Materials

Ethyl alcohol, ferrocene, thiophene, sulfuric acid (H_2_SO_4_), hydrochloric acid (HCl) and sodium hydroxide (NaOH) were obtained from Sinopharm Chemical Reagent Shanghai Co., Ltd. (Shanghai, China). Titanium butoxide (Ti(OH_9_C_4_)_4_), n-hexane and RhB were purchased from Aladdin Reagent Database Inc. (Shanghai, China). All chemicals were of analytical grade and utilized without further purification.

### 2.2. Fabrication of SCTF

CNT films with an area of meter level and a thickness of ca. 8 μm were manufactured by the FCCVD method [18,35]. TiO_2_ nanowires were coupled facilely onto porous CNT films via a hydrothermal strategy as reported in previous works [36,37]. Specifically, the pristine CNT films were cut into identical circles (Ø 5.5 cm) and then engraved by laser drilling. The porous CNT films (10 mg) were eluted with 1 M H_2_SO_4_ to remove the iron catalysts, followed by washing with deionized (DI) water until reaching a neutral solution. Subsequently, the treated porous CNT films were immersed into a 50 mL solution of Ti(OH_9_C_4_)_4_ and n-hexane with a volume ratio of 1:9 for 5 min at room temperature, followed by taking out and exposure in air for 10 min to enable the n-hexane volatilization and hydrolysis of Ti(OH_9_C_4_)_4_ into seed crystals. 

Afterward, the obtained composite films were mixed with 80 mL NaOH aqueous solution (10 M) and 0.5 mL Ti(OH_9_C_4_)_4_. The mixture was, then, transferred into a 100 mL Teflon-line stainless steel reactor and maintained at 200 °C, and the amount of TiO_2_ nanowires generated on porous CNT films could be regulated by changing the reaction time. After cooling naturally to room temperature, the resultant films were washed with 0.1 M HCl and DI water in sequence for several times, followed by annealing at 500 °C for 3 h, thus, eventually achieving SCTFs. The mass fractions of CNTs in the SCTFs were 2%, 2.5%, 3.5% and 5%, respectively, and the final products were named SCTF-2, SCTF-2.5, SCTF-3.5 and SCTF-5.

### 2.3. Characterization

Scanning electron microscope (SEM) images were collected using a field emission scanning electron microanalyzer (Apreo C HiVac, Thermo Fisher Scientific, Waltham, MA, USA) at an acceleration voltage of 10 kV. X-ray diffraction (XRD, Bruker Inc., karlsruhe, Germany) patterns were recorded on a Bruker AXS X-ray diffractometer (Bruker Inc., karlsruhe, Germany) using a Cu Kɑ radiation generator (1.5406 Å, 40 kV and 40 mA). Raman spectra were obtained by a Renishaw RM3000 Raman microscope (Renishaw Inc., London, UK) with a 633 nm laser source. UV-vis diffuse reflectance spectra (DRS) were measured using a Hitachi U-4100 spectrophotometer (Hitachi Limited, Tokyo, Japan) with BaSO_4_ as a reflectance reference. The contact angle was determined by a Drop Shape Analyzer (DSA100, KRÜSS, Hamburg, GmbH). The tensile stress–strain tests were carried out on an Instron 3365 equipped with a fine force detector at a tensile rate of 0.5 mm per minute.

### 2.4. Photocatalytic Degradation Procedure

The photocatalytic activity of as-fabricated SCTFs was evaluated by the degradation of RhB. A 300 W Xe lamp (Shaoxing Changtuo Imp. Exp. Co., Shaoxing, China) with a wavelength range of 420–800 nm and light intensity of 100 mW cm^−2^ was used as the simulated solar light source. The light intensity of sunlight was 45 mW cm^−2^. Briefly, 12 mg of photocatalyst was immersed into 100 mL of RhB aqueous solution (10 mg L^−1^). The obtained suspension was placed for 30 min in the dark to attain the adsorption–desorption equilibrium and then exposed to visible light. At a given interval of 10 min, 3 mL of RhB aqueous solution was taken out, and its concentration was measured with a UV-vis spectrophotometer (Model lambda 35, PerkinElmer) at a maximum absorption wavelength of 553 nm.

## 3. Results and Discussion

### 3.1. Fabrication and Characterization of SCTF

**Figure 1:**
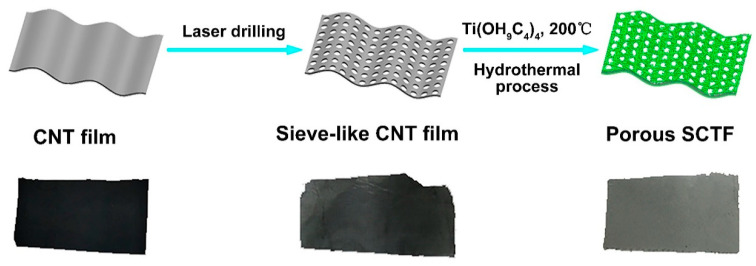
The schematic procedure of fabricating sieve-like carbon nanotube (CNT)/TiO2 nanowire film (SCTF) and the corresponding films obtained at each step.

The procedure for the SCTF fabrication is schematically illustrated in Figure 1. The flexible CNT film was treated by laser drilling to form well-organized holes with a pore size on the micrometer level. Subsequently, TiO_2_ nanowires were coupled with the resultant sieve-like CNT film via hydrothermal treatment in an aqueous solution of Ti(OH_9_C_4_)_4_ at 200 °C, eventually forming a porous SCTF. From the optical images of films, the film featured no significant destruction in the structure (e.g., fractures and cracks) and its color changed from black to gray owing to the pore formation and TiO_2_ modification on the CNT film during the process of laser drilling and hydrothermal treatment.

**Figure 2:**
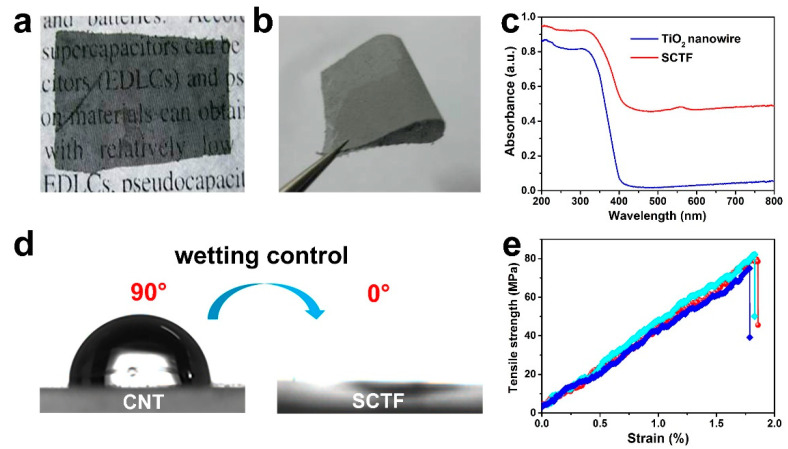
(**a**,**b**) Digital photographs of SCTF. (**c**) UV-vis DRS of a TiO_2_ nanowire and SCTF. (**d**) Photograph of a water droplet standing on a sieve-like CNT film and SCTF. (**e**) Tensile stress–strain curves of SCTFs.

Figure 2a presents the optical photograph of an SCTF placed on a piece of paper with certain words. A high definition of these words below the film was observed, implying excellent transparency of the SCTF, which was propitious to the light penetration into the interior of film when exposed to solar irradiation. As envisioned in Figure 2b, even under bending with an angle up to 180°, SCTF displayed exceptional flexibility without any local damage generated, thus, confirming that the inner structure of film was well maintained. The light harvesting and absorption ability of the TiO_2_ nanowire and SCTF was examined using UV-vis DRS. 

In contrast to the neat TiO_2_ nanowire, SCTF featured significantly enhanced UV-vis light absorption, especially within the visible light range (Figure 2c), signifying that coupling a TiO_2_ nanowire to a sieve-like CNT film would be favorable for improving both the visible light absorption and photocatalytic activity. Static water-droplet contact angle measurements were used to characterize the surface wettability. 

As illuminated in Figure 2d, the contact angle at the surface of the sieve-like CNT film was 90°, and this was drastically reduced to 0° at the surface of the SCTF, thereby, proving that a significantly enhanced surface wettability of SCTF originated from the introduction of TiO_2_ nanowire with abundant hydrophilic hydroxyl groups, which would be beneficial to the polluted water diffusion throughout the entire film when served as a photocatalyst. Additionally, the as-prepared SCTFs featured a satisfactory mechanical property with tensile strength of 75–82 MPa and fracture elongation of 1.79–1.86% (Figure 2e), which was critical and essential to their use in the continuous-flow photodegradation of polluted water.

**Figure 3:**
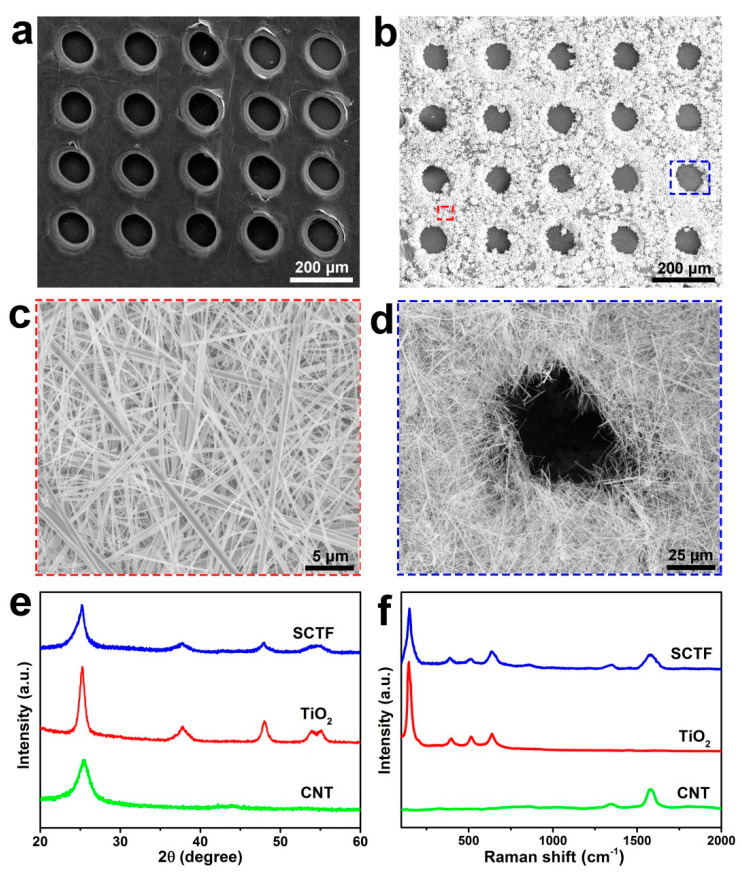
SEM images of a sieve-like CNT film (**a**) and SCTF at low and high magnifications (**b**–**d**). XRD patterns (**e**) and Raman spectra (**f**) of CNT, TiO_2_ and SCTF.

The microstructure of sieve-like CNT films and SCTFs was characterized through SEM. The sieve-like CNT film displayed a relatively flat surface and consisted of abundant, well-ordered and uniform mesopores with pore diameter of ca. 100 µm and pore interval of ca. 70 µm (Figure 3a). In contrast, SCTF displayed an obvious difference (Figure 3b). The massive needle-like substances were homogeneously attached to the surface of SCTF. Additionally, note that the pore size was reduced from 100 to 70 µm, however, they maintained a nearly circular shape. 

The magnified SEM image corresponding to the red frame-marked region in b was featured by numerous interlaced fibers with an average diameter of ca. 500 nm (Figure 3c), while the SEM image corresponding to the blue square region in b expressly presented a pore with a rough edge encircled by a network structure (Figure 3d). The homogeneous distribution of nanowires and high porosity on SCTF made it favorable for the continuous-flow photodegradation of organic pollutants when served as photocatalyst due to effective photoexcitation and convenient liquid penetration. 

The composition of the SCTFs was examined using XRD and Raman spectra, and, for a comparison, the XRD and Raman spectra of the CNTs and TiO_2_ were also analyzed. As seen in Figure 3e, all characteristic peaks of the CNTs and TiO_2_ appeared in the XRD pattern of the SCTFs, as expected. For instance, these sharp and intensive peaks at 2θ of 25.23°, 37.91°, 48.04°, 53.81° and 55.14° corresponding to the reflections from the (101), (004), (200), (105) and (211) crystalline planes of anatase TiO_2_, respectively, as well as a relatively broad peak of the CNTs at 25.51°. The Raman analysis of SCTFs presented D and G bands corresponding to CNTs at 1338 and 1580 cm^−1^, as well as characteristic peaks of anatase TiO_2_ at 147, 397, 515 and 638 cm^−1^ (Figure 3f). Both the XRD and Raman spectra proved that anatase TiO_2_ nanowires were successfully combined with a porous CNT film to form the composite SCTF.

### 3.2. Photocatalytic Performance of SCTF

#### 3.2.1. Optimization of SCTF

**Figure 4:**
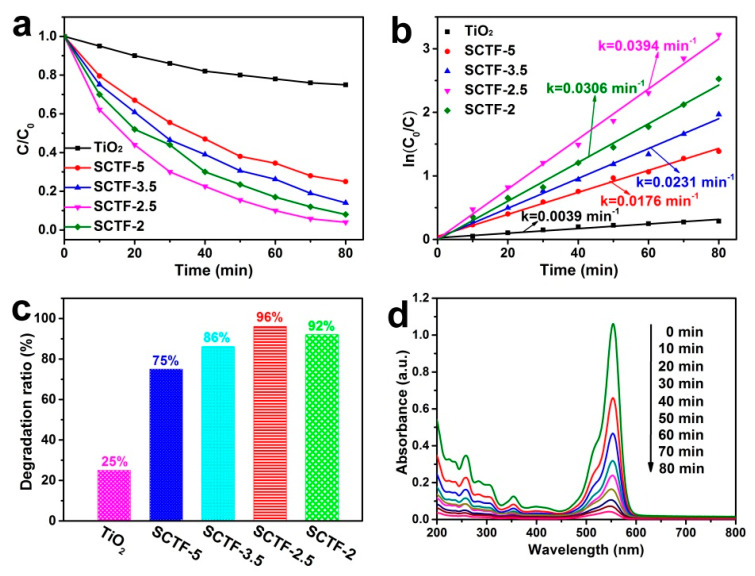
Photocatalytic degradation curves (**a**), kinetic curves (**b**) and degradation ratios (**c**) of RhB for different photocatalysts under simulated solar irradiation. (**d**) Absorption spectra of RhB with SCTF-2.5 as photocatalyst in different reaction times under simulated solar irradiation.

The photocatalytic activity of the as-fabricated samples was evaluated through the degradation of RhB under visible light irradiation. Prior to irradiation, the RhB solutions containing photocatalysts were kept in the dark under continuous stirring for 30 min to reach the adsorption–desorption equilibrium. Figure 4a presents the C/C_0_ profile as a function of the irradiation time, where C and C_0_ refer to RhB concentrations at a given time and the initial time before irradiation, respectively. For pure TiO_2_, the RhB was slowly degraded, with a degradation ratio of only 25% within 80 min, manifesting its poor photocatalytic performance. 

In contrast, all the SCTF heterojunction photocatalysts displayed a significantly enhanced photocatalytic activity toward the removal of RhB as expected. The degradation ratio of RhB reached 75%, 86%, 96% and 92% for SCTF-5, SCTF-3.5, SCTF-2.5 and SCTF-2, respectively (Figure 4c). SCTF-2.5 featured the most pronounced photocatalytic activity, which was approximately four-times higher than that of the pure TiO_2_ sample. At high TiO_2_ loading, a decline in the degradation ratio occurred owing to the shading effect and the aggregation of TiO_2_ nanowires, which could hinder the light transmission to the surface of catalysts and restrict the efficient light absorption. 

As seen in Figure 4b, the time-dependent photodegradation behavior of RhB obeyed a pseudo-first-order kinetic model: ln(C_0_/C) = kt (where t is the irradiation time and k is the rate constant) [38,39]. The calculated k value for SCTF-2.5 was 0.0394 min^−1^, which was 10.10-, 2.24-, 1.71- and 1.29-times higher than pure TiO_2_, SCTF-5, SCTF-3.5 and SCTF-2, respectively, and was also substantially higher as compared to most of the previously reported values for other carbon-based photocatalysts [40,41,42]. The above findings demonstrate that the loading of TiO_2_ nanowires to a porous CNT film was beneficial for enhancing its photocatalytic performance toward RhB degradation, and SCTF-2.5 exhibited much better photocatalytic activity, which was used for the following photodegradation measurements. 

Figure 4d shows the changes in the typical time-dependent absorption spectra of RhB during the photodegradation process. The maximum absorption peak of RhB, centered at 553 nm, gradually decreased with the increasing irradiation time and almost disappeared after simulated solar irradiation for 80 min, implying that most of RhB could be degraded in the presence of SCTF-2.5. For comparison, the photodegradation of RhB was also conducted under the identical conditions with no photocatalyst and only a CNT film. 

As envisioned in Figure S1, after irradiation for different times, the absorbance of RhB remained almost unchanged in the absence of a photocatalyst and displayed a slight decline in the presence of the CNT film, which revealed poor photodegradation of RhB, thus, further proving the prominent photocatalytic activity of SCTF-2.5 to degrade RhB. Figure S2 compares the photocatalytic behavior of RhB under natural and simulated solar irradiation. RhB was effectively degraded under natural solar irradiation, despite the relatively lower degradation ratio at the same irradiation time and rate constant as compared to that under simulated solar irradiation due to the low light intensity of the sun, which suggests a potential application of SCTF-2.5 to RhB degradation in real-world situations.

#### 3.2.2. Continuous-Flow Photodegradation

**Figure 5:**
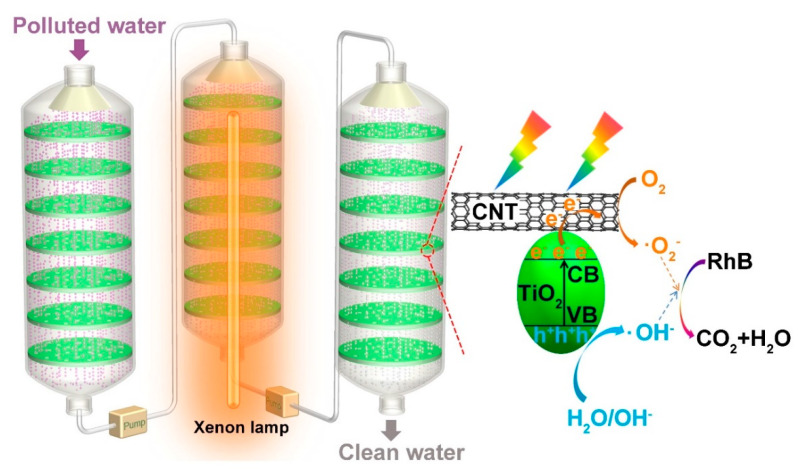
Schematic diagram of the continuous-flow photodegradation and photocatalytic degradation mechanism of RhB on SCTF-2.5.

The photocatalytic oxidation of organic pollutants to carbon dioxide (CO_2_) in a continuous and scalable way is becoming vital concern for the efficient treatment of polluted water. To verify the feasibility of SCTF for continuous-flow photodegradation, a single-inlet–outlet plate column-like photoreactor was elaborately designed and assembled to carry out the continuous-flow photooxidation of RhB. Figure 5 shows a schematic illustration of the ready-to-use continuous-flow device connected by three photoreactors in a series. 

The photoreactor was constructed by consecutively fixing SCTF-2.5 (Ø 5 cm) into a sealed transparent glass vessel with an ca. 3 cm distance between the SCTF-2.5 photoreactors. The aqueous RhB solution was sprayed into the photoreactor through the inlet, which simultaneously allowed the input of air. The photodegradation was operated in a continuous-flow mode with a continuous RhB inflow from the bottom of the front photoreactor to the top of the latter photoreactor via syringe pumps. 

To realize the photodegradation of RhB to CO_2_ in the continuous-flow device, an optimized condition was used after multiple trial experiments. Specifically, the device was fed with aqueous RhB solution (<2 mg L^−1^) at a flow rate of 2 mL min^−1^, and air was introduced under one bar of pressure. The photooxidation reaction of RhB was performed at room temperature with simulated solar irradiation, and the products were collected from the outlet of the device for analysis at certain time intervals. 

**Figure 6:**
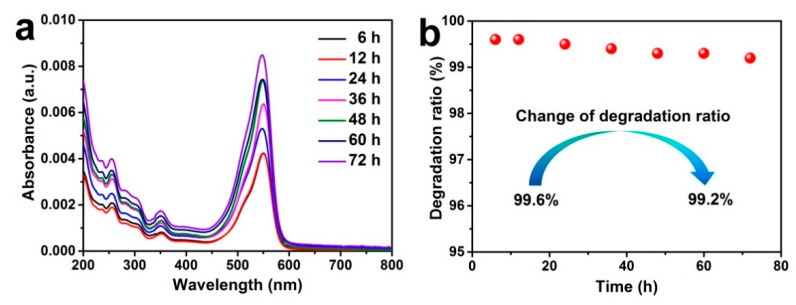
UV-vis spectra (**a**) and the degradation ratios (**b**) of the RhB solution for different times during the continuous-flow photodegradation under simulated solar irradiation.

As seen in Figure 6, when the time interval of sampling varied from 6 to 72 h, very low absorbance and a slight change in absorbance were observed (Figure 6a), and the degradation ratio of RhB maintained a high level ranging from 99.2–99.6% (Figure 6b), adequately demonstrating that the as-fabricated device was feasible for the continuous-flow photodegradation of RhB under visible light irradiation with a high degradation ratio and long-term stability. Based on the obtained results, a possible mechanism for the photocatalytic degradation of RhB in the continuous-flow device was proposed. 

Under simulated solar irradiation, TiO_2_ nanowires coupled into a porous CNT film in the SCTF became photoexcited to produce electrons (e^−^) and holes (h^+^). The photogenerated electrons could be transferred to the CNT, which would be further captured by O_2_ to form ^•^O_2_^−^. The positively charged holes in the valence band were able to react with H_2_O/OH^−^ to generate ^•^OH. ^•^O_2_^−^ and ^•^OH as powerful oxidants effectively degraded RhB into CO_2_ and H_2_O. 

## 4. Conclusions

In summary, to further extend and boost the photocatalytic application of CNT films, a flexible and robust SCTF with a tensile strength of 75–82 MPa for the continuous degradation of polluted water was fabricated via a facile coupling of a TiO_2_ nanowire onto a laser-drilling CNT film. The resulting hybrid film demonstrated the distinctive advantages of good transmittance and convenient visible light absorption, thus, guaranteeing high photosensitivity and effective photoexcitation. SCTF revealed unique structural features with abundant, well-ordered and uniform mesopores (ca. 70 µm in diameter) as well as the homogenous distribution of TiO_2_ nanowires (ca. 500 nm in diameter), thereby, enabling channels for efficient solution infiltration through the film during a continuous degradation of pollutants. 

In contrast, SCTF-2.5 featured a high photocatalytic performance for RhB degradation with a degradation ratio of 96% and rate constant of 0.0394 min^−1^. The continuous-flow photodegradation device assembled with SCTF-2.5 achieved a high degradation ratio (99.6%) of RhB and long-term stability (99.2% retention after working continuously for 72 h) under simulated solar irradiation.

## Data Availability

The data presented in this study are available on request from the corresponding author.

## Supplementary Materials

The following are available online at https://www.mdpi.com/article/10.3390/nano11051335/s1, Figure S1: Photodegradation of RhB with no photocatalyst and only CNT film, Figure S2: Comparison of RhB degradation under natural and simulated solar irradiation.
