# Supplementary material for: Sieve-Like CNT Film Coupled with TiO2 Nanowire for High-Performance Continuous-Flow Photodegradation of Rhodamine B under Visible Light Irradiation
Source: Nanomaterials (Basel). 2021 May 19;11(5):1335. doi: 10.3390/nano11051335 (PMC8159084; doi:10.3390/nano11051335)
Supplement: Supplementary file 1 [file nanomaterials-11-01335-s001.zip › nanomaterials-1230764-supplementary.pdf]

Supporting information

Sieve-Like CNT Film Coupled with TiO_2 Nanowire for High-Performance Continuous-Flow Photodegradation of Rhodamine B under Visible Light Irradiation

Zhengpeng Yang [1], Xiaoting Lv [1,2], Xuqing Liu [3], Shengmin Jia [1,2], Yongyi Zhang [2,4,*], Yingying Yu [2,*], Chunjing Zhang [1] and Dandan Liu [2,*]

[1] Henan Key Laboratory of Materials on Deep-Earth Engineering, School of Materials Science and Engineering, Henan Polytechnic University, Jiaozuo 454003, China

[2] Key Laboratory of Multifunctional Nanomaterials and Smart Systems, Advanced Materials Division, Suzhou Institute of Nano-Tech and Nano-Bionics, Chinese Academy of Sciences, Suzhou 215123, China

[3] Department of Materials, The University of Manchester, Oxford Road, Manchester M13 9PL, UK

[4] Division of Nanomaterials and Jiangxi Key Lab of Carbonene Materials, Suzhou Institute of Nano-Tech and Nano-Bionics, Nanchang, Chinese Academy of Sciences, Nanchang 330200, China

* Corresponding authors at: Suzhou Institute of Nano-Tech and Nano-Bionics, Chinese Academy of Sciences, Suzhou 215123, PR China.

E-mail addresses: yyzhang2011@sinano.ac.cn (Y.Y. Zhang), yyyu2019@sinano.ac.cn (Y.Y. Yu), ddliu2015@sinano.ac.cn (D.D. Liu).

Supplementary Figures

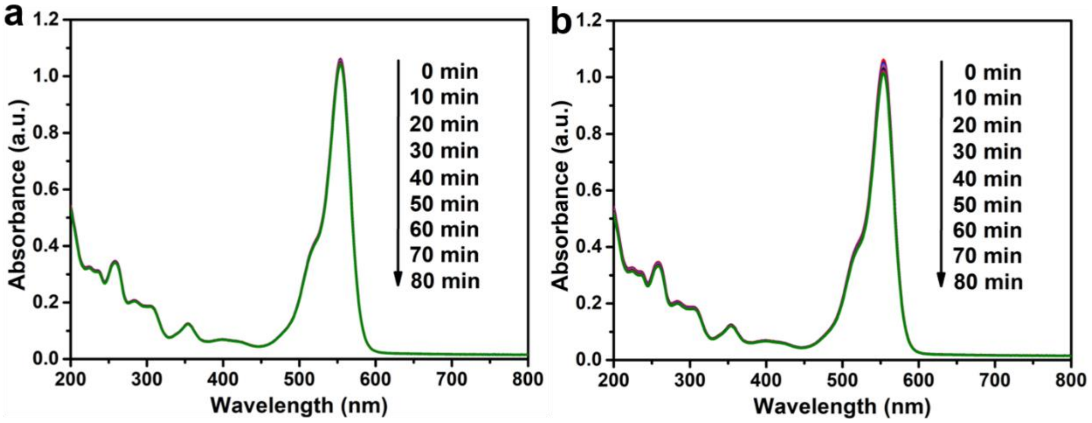

Figure S1. Absorption spectra of RhB with no photocatalyst (a) and only CNT film (b) in different reaction time under simulated solar irradiation.

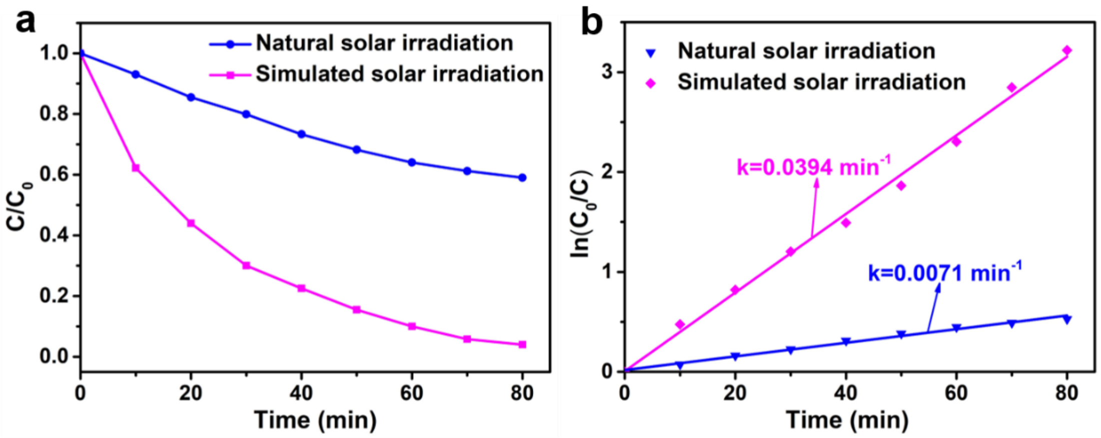

Figure S2. Photocatalytic degradation curves (a) and kinetic curves (b) of RhB with SCTF-2.5 as photocatalyst under natural and simulated solar irradiation.
